# Pillars and Gaps of S-Nitrosylation-Dependent Epigenetic Regulation in Physiology and Cancer

**DOI:** 10.3390/life11121424

**Published:** 2021-12-17

**Authors:** Luisa Salvatori, Francesco Spallotta, Carlo Gaetano, Barbara Illi

**Affiliations:** 1 Institute of Molecular Biology and Pathology—National Research Council (IBPM-CNR), 00185 Rome, Italy; luisa.salvatori@cnr.it; 2 Institute of System Analysis and Computer Science “A. Ruberti”—National Research Council (IASI-CNR), 00185 Rome, Italy; francesco.spallotta@cnr.it; 3 Istituti Clinici Scientifici Maugeri—IRCCS, 27100 Pavia, Italy; carlo.gaetano@icsmaugeri.it

**Keywords:** nitric oxide, S-nitrosylation, epigenetics, gene expression regulation, cancer, post-translational modifications

## Abstract

Nitric oxide (NO) is a diffusible signaling molecule produced by three isoforms of nitric oxide synthase, which release NO during the metabolism of the amino acid arginine. NO participates in pathophysiological responses of many different tissues, inducing concentration-dependent effect. Indeed, while low NO levels generally have protective effects, higher NO concentrations induce cytotoxic/cytostatic actions. In recent years, evidences have been accumulated unveiling S-nitrosylation as a major NO-dependent post-translational mechanism ruling gene expression. S-nitrosylation is a reversible, highly regulated phenomenon in which NO reacts with one or few specific cysteine residues of target proteins generating S-nitrosothiols. By inducing this chemical modification, NO might exert epigenetic regulation through direct effects on both DNA and histones as well as through indirect actions affecting the functions of transcription factors and transcriptional co-regulators. In this light, S-nitrosylation may also impact on cancer cell gene expression programs. Indeed, it affects different cell pathways and functions ranging from the impairment of DNA damage repair to the modulation of the activity of signal transduction molecules, oncogenes, tumor suppressors, and chromatin remodelers. Nitrosylation is therefore a versatile tool by which NO might control gene expression programs in health and disease.

## 1. Introduction

The free radical nitric oxide (NO), identified some decades ago as the endothelium-derived relaxing factor [1], is a short-lived (having a half-life of a few seconds), gaseous signaling molecule highly diffusible across cell membranes. Nowadays, it has been widely demonstrated that NO plays important roles in many biological processes, and therefore, its deregulation might participate in pathological disorders, including cancer. The focus of this review is to specifically highlight the role of S-nitrosylation in mediating NO-dependent epigenetic effects in physiological and oncological contexts. Furthermore, despite the great amount of information now available, lacunae in the field still exist and are also discussed.

### 1.1. NO Chemistry

NO is produced by a family of nitric oxide synthases (NOS) that release NO during the conversion of arginine to citrulline [2]. NO can exert its biological effects through many different chemical reactions, thus generating a wide range of signaling pathways. Typically, NO acts through its receptor, soluble guanylate cyclase (sGC), leading to the synthesis of the second messenger cGMP. The subsequent activation of cGMP-dependent kinases induces a cascade of protein phosphorylations, which allow the signal transduction to start [3]. In addition, through the reaction with O_2_ and O_2_^−^, NO can be metabolized to form reactive nitrogen species (RNS), including the powerful nitrosating agent dinitrogen trioxide (N_2_O_3_) and peroxynitrite (ONOO^−^), a potent cytotoxic oxidant able to induce DNA damage [4,5]. Nitrosative stress may induce protein post-translational modifications mainly through protein tyrosine (Tyr) nitration, which signals cellular damage. However, Tyr-nitration might act also as a free radical process relying on the addition of a nitro group (-NO_2_) to the position 3 of the Tyr phenolic ring with the occurrence of the formation of 3-NT [6]. NO can also act through covalent binding to sulfur atoms on proteins and inorganic compounds to form S-nitrosothiols (SNOs). This modification, affecting protein function, stability, and localization [7], is the focus of the present review, and therefore, its relevance will be extensively discussed below.

Through the reaction with diverse molecules, NO participates in pathophysiological responses of many different tissues with frequently contradictory biological effects [8,9]. Indeed, NO-induced effects depend on different conditions, such as NO concentration, its rate of diffusion, the presence of potential reactants and targets, as well as their distance [8]. Among all these variables, the different cellular responses mostly depend on NO concentration, which determines NO chemistry. Indeed, under normal physiological conditions, low NO concentrations, such as those generated by neural and endothelial NOS (NOS2 and NOS3) isoforms, are generally produced through fast chemical reactions in which NO directly reacts with the target molecule, resulting in cytoprotective effects mediated by antioxidant mechanisms [8,10]. Differently, at much higher NO concentrations (such as those produced via the inducible NOS1 isoform), indirect effects occur through generation of RNS, which subsequently react with biological targets, serving cytotoxic/cytostatic functions [8,10].

### 1.2. NO in Cancer 

NO has wide actions in tumor biology, including modulation of apoptosis, cell cycle, DNA integrity, mitogenic pathways, invasion, and angiogenesis. However, NO may induce both protective tumor-promoting actions and anti-proliferative effects through the inhibition of oncogenic pathways or the activation of tumor-suppressor genes [11]. As in healthy cells, the dual role of NO in cancer is dose-dependent; in fact, NO contributes to cancer progression at low concentrations, whereas it is detrimental for tumor survival at high concentrations [12,13]. Indeed, NO overproduction acts as a pro-apoptotic player activating the caspase family of proteases through mitochondrial cytochrome c release and up-regulation of p53. On the contrary, low NO concentrations are anti-apoptotic [14,15,16].

Of particular importance is the observation that NO might induce direct modifications of DNA, inhibit DNA repair enzymes, and promote DNA strand breaks and mutations through RNS [17,18,19]. Peroxynitrite usually leads to an increased level of DNA damage complexity consistent with its higher reactivity [20]. In this context, DNA-damaging agents determine a temporary cell cycle arrest, but if the damage is too extensive, the cells undergo apoptosis [13]. NO affects both cell proliferation and p53 expression [21,22], which senses DNA damage, thus affecting cell cycle progression and apoptosis.

The role of NO in tumor progression includes the activation of mitogenic pathways. Indeed, among others, NO activates the epidermal growth factor receptor (EGFR), the extracellular signal-regulated kinase (ERK), mTOR, Ets-1, and Wnt/β-catenin signaling, resulting in increased proliferation, angiogenesis, migration, and invasion [23,24,25,26]. However, NO negatively regulates JNK and Akt pathway as well as the expression of the oncogene N-Myc and blocks ERK1/2 activity through direct modification of H-Ras [27,28,29,30].

The epithelial-to-mesenchymal transition (EMT) is pivotal to cancer cells to migrate and spread throughout the body. Low NO levels promote cell migration and invasion in diverse tumor cell models [23,31,32]. Conversely, high NO concentrations reverse EMT and the invasive phenotype of cancer cell lines [33,34,35].

Cancer progression and metastasization also depends on angiogenesis, which supports tumor growth and allows cancer cell to reach tissue districts far from the primary tumor site. NO participates in regulation of angiogenesis, showing both pro-angiogenic properties and anti-angiogenic effects [36]. Indeed, NO promotes angiogenesis by inducing endothelial differentiation, increasing tumor vasculature permeability, promoting tumor blood flow, inducing the production of pro-angiogenic factors, and inhibiting antiangiogenic factors [37,38].

Cancer stem cells (CSC) are recognized as crucial component of the tumor, being involved in tumor initiation, progression, metastasis, and therapy resistance [39]. It has been shown that NO can support stemness-related signaling pathways and CSC phenotype in a variety of tumors [40,41].

Further, NO plays a dual role in regulating the immune system response to non-self-antigens expressed on the surface of cancer cells [42]. Indeed, it has been widely reported that NO has immunosuppressive properties, such as inhibition of immune cell chemotaxis, adhesion, and infiltration, as well as direct inhibition of T-cell proliferation and function [43,44,45,46]. Therefore, NO might reduce the immune response against tumors promoting tumor growth and spread. However, the induction of macrophage-dependent NO production within tumor microenvironment contributes to the success of immunotherapy, suggesting NO as an adjuvant for this highly promising anti-tumor therapeutic route. [43,47,48,49,50].

### 1.3. S-Nitrosylation

S-nitrosylation (therein referred as nitrosylation) is a reversible and highly regulated in time and space, post-translational modification which couples a NO moiety to a cystein (Cys) thiol, generating SNOs. The NO moiety might be provided by NO directly or by metal-containing NO. A major source of NO is the heme iron-nitrosyl species (FeNO), whose formation depends on NOS, low-mass SNOs or nitrite [51]. Other sources of NO are represented by dinitrosyl–iron complexes (DNICs) [52]. Specificity is one of the most important characteristics of this chemical phenomenon. Indeed, despite all proteins possess many potential Cys residues target of nitrosylation, only one or few Cys are effectively nitrosylated upon physiological or pathological stimuli. Besides the interaction with NOS, which puts in close proximity the source of NO and the target protein [53,54,55], the determinants of this specificity are electrostatic interactions, thiol accessibility or reactivity regulated by allosteric modulators, and hydrophobic compartmentalization.

One of the motifs suggested for nitrosylation is the acid-base motif, where the Cys flanking sequences are composed by acidic (Asp, Glu) and basic (His, Lys, Arg) amino acids [56]. The details of acid-base catalysis of nitrosylation/de-nitrosylation are described in reference [57].

Allosteric nitrosylation might depend on ions (Mg^++^, Ca^++^, H^+^) or O_2_-related species, which might cause protein conformational changes favoring protein S-nitrosylation/de-nitrosylation [58,59]. Prominent examples of allosteric nitrosylation are represented by hemoglobin and the ryanodine receptor/calcium release channel 1 (RyR1). Both molecules are sensitive to O_2_ and dynamically change their conformation according to O_2_ tension. Hemoglobin response to O_2_ binding to heme irons results in NO binding and nitrosylation of Cysβ93 with the formation of SNO-hemoglobin. Consistently, deoxygenation reverses this conformational switch and allows NO release. This reversible conformational transition allows hemoglobin to sense tissues O_2_ requirement. Indeed, SNO-hemoglobin leads to vessels contraction and decreases blood perfusion, whilst deoxygenated hemoglobin acts in the opposite way [58]. RyR1 senses tissue pO_2_, which is lower than ambient pO_2_. Physiological O_2_ tension controls the redox state of 6–8 out of five thiols in a single RyR1subunit, regulating nitrosylation of a single-channel thiol. The conformational change induced by physiological pO_2_ creates a hydrophobic compartment concentrating NO and O_2_, boosting the generation of SNO moieties. This phenomenon does not occur in ambient pO_2_ [59].

NO oxides (NO_2_, NO_2_NO_3,_ NO_2_NO_4_), the reaction products of NO with O_2_ or with superoxide, might accumulate in cell membranes [60], leading to nitrosylation via redox-based mechanisms [61], and some nitrosylated proteins possess their target Cys in a juxtamembrane zone [62]. Hydrophobic regions within proteins, due to tertiary structure and protein–protein interactions, might also promote nitrosylation [63]. In general, a hydrophobic environment may retain radical species and impairs SNOs hydrolysis.

The catalysts of nitrosylation/de-nitrosylation are a variety of enzymes or protein-bound transition metals. Superoxide dismutase (SOD) catalyzes the nitrosylation of hemoglobin [64] and the Cu^2+^-containing ceruloplasmin the S-nitrosylation of S-nitrosoglutathione (GSNO) from free NO [65] and that of heparin-sulphate proteoglycan glypican 1 [66]. Protein-bound transition metals and flavins may catalyze transnitrosylation of proteins, the transfer of a NO moiety from SNOs to other thiols in other targets, in a sort of auto-nitrosylation process [67,68,69].

SNO-based defense signals must be switched off to protect cells from persisting nitrosative stress. Most of SNO proteins are expected to be de-nitrosylated by glutathione (GSH), the most abundant intracellular source of thiols, by trans-nitrosylation reactions. De-nitrosylation might occur upon catalytic intervention of thioredoxin (TRX) [70], S-nitrosogluthatione (GSNO) reductase (GSNOR) [71,72], and protein disulfide isomerase (PDI) [73]. These are the major enzymes catalyzing protein de-nitrosylation. TRX de-nitrosylation occurs through a trans-nitrosylation process, which involves Cys32 and Cys35, with the generation of a disulfide ring structure, which releases nitroxyl (HNO). Further nitrosylation of Cys62, Cys69, or Cys73 might also occur. TRX-SNO is reduced by TRX reductase in the presence of NADPH [70]. Alcohol Dehydrogenase III (ADHIII), now recognized as GSNOR, catalyzes the production of glutathione sulfinamide in the presence of NADH and oxidized glutathione starting from GSNO [73]. PDI is characterized by two active subunits, namely subunit a and subunit a’. During de-nitrosylation, one thiol of PDI active subunit a undergoes trans-nitrosylation in the presence of GSNO. The following catalytic reactions, which are characterized by the formation of stable and unstable intermediates, finally produce an oxidized PDI subunit a and NO [73].

## 2. Epigenetics of S-Nitrosylation

Since 2008, the epigenetic functions of NO were merely speculative. Thereafter, a great deal of information has been accumulated underpinning the epigenetic control of gene expression as one of the downstream effects of NO and NO-related post-translational modifications, such as tyr-nitration and nitrosylation. The presence of NOS within the nucleus [74,75,76] strongly suggests a direct effect of NO in controlling the expression of genes by chemical modification of both DNA and histones. This intriguing NO-dependent route to gene transcriptional regulation is still under investigation, whilst indirect mechanisms are now well established.

### 2.1. Direct S-Nitrosylation of DNA and Histones

NO direct regulation of chromatin in terms of architecture and accessibility to transcriptional machineries is still under investigation. NO-dependent modification of DNA and histones is a quite unexplored field. Most of the knowledge regarding NO regulation of chromatin is related to histone nitration. This is a well-documented phenomenon, established both in vitro [77] and in vivo [78]. The functional role of this modification seems to be related to chromatin compaction and protection of the DNA from oxidative damage. Of note, nitrated histones are found in many human pathological contexts, from autoimmune diseases [79] to liver injury [78], suggesting these specifically modified histone species as novel potential clinical biomarkers.

The “genome-wide oscillation hypothesis” is one of the most fascinating mechanisms proposed of a direct NO action on chromatin. In the presence of metal ions (e.g., Fe^++^), NO might react with thiols. According to this hypothesis, the resulting DNICs release nitrosonium (NO^+^), accounting for nitrosylation within chromatin. Both thiols and metal ions are largely represented within the nucleus and chromatin. Indeed, metal ions are allowed to react with the DNA phosphate backbone and bases within the minor and the major grooves of the double helix, whose stability is largely influenced by metal-mediated redox changes [80,81]. Thiol nuclear source is represented by histones, which contain Zn-finger modules [82], transcription factors, and chromatin remodelers. The nuclear triad composed of NO, thiols, and metal ions might account for the oscillatory assembly/disassembly of protein complexes, leading to cycles of transcriptional activity, the so-called “genome-wide oscillation” [83]. As discussed above, whilst histone nitration is a well-documented phenomenon [47,48,49,50], only recently nitrosylation of histone H2B and H3 has been clearly demonstrated in *Trypanosoma cruzi* [84]. However, the targeted cysteines (Cys124 and Cys126) are not conserved in higher eukaryotes in which histone nitrosylation is still an uncovered entity.

### 2.2. S-Nitrosylation-Dependent Indirect Epigenetic Mechanisms

Several papers deal with nitrosylation of kinases along signalling pathways, transcription factors (TFs), and transcriptional co-regulators. We will focus on nitrosylation-dependent regulation of molecules directly involved in the regulation of the chromatin landscape, that is, TFs and chromatin remodeling enzymes, as this represents a well-documented key route leading to gene expression regulation by NO (Table 1).

#### 2.2.1. S-Nitrosylation of Transcription Factors 

**Figure 1:**
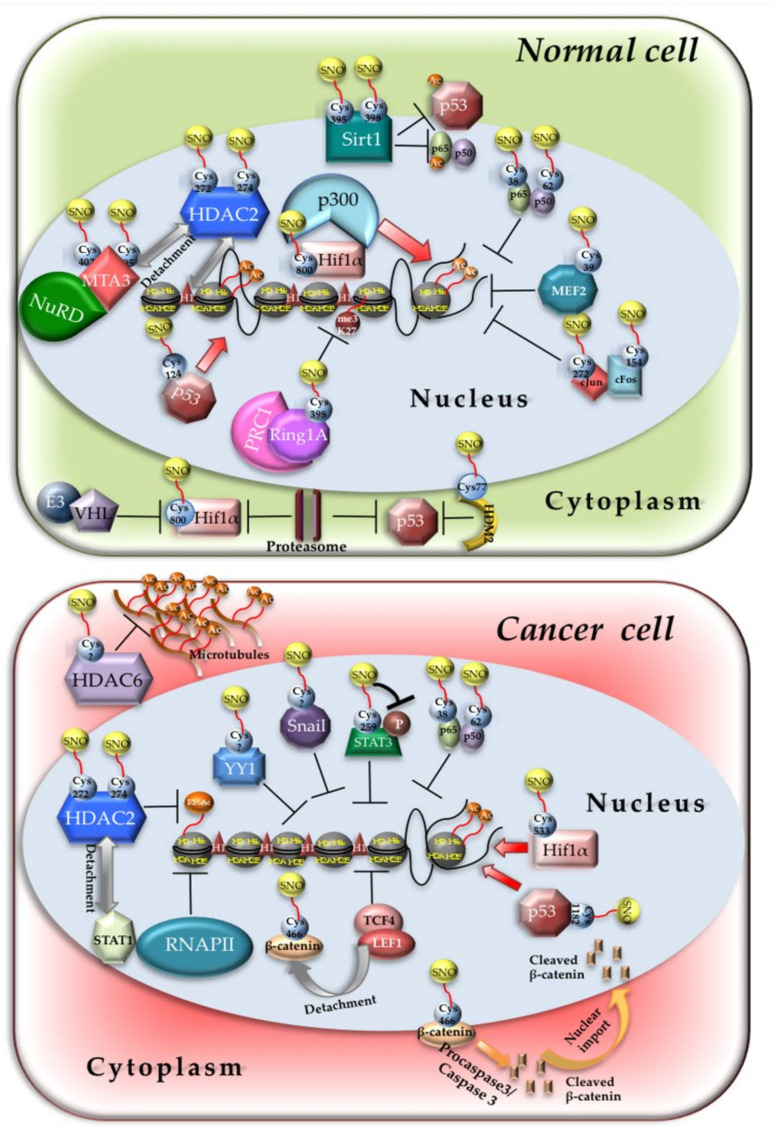
Nitrosylation of chromatin regulators in normal and cancer cells. Schematic representation of nitrosylation-dependent effects on TFs and chromatin remodelers functions in normal (**top**) and cancer cells (**bottom**) described throughout the manuscript. Grey arrows indicate protein detachment either from chromatin or protein partners; red arrows indicate TFs and remodeling enzymes recruitment/activation.

NO-dependent modification of TFs is a common conserved mechanism that the organisms use to regulate their response to extracellular and intracellular NO. The related mechanisms of action rely on changes in protein–protein interactions and protein–DNA interactions. The impact of nitrosylation on TFs activity mainly depends on the location of the target Cys residues. If the Cys is located within the DNA binding domain, usually nitrosylation inhibits TF activity [85]. If nitrosylation affects a Cys within an interaction domain with co-regulators, it might either improve or decrease reciprocal binding and transcription. Many TFs are nitrosylated in mammalian cells. Nitrosylation of HIF1α on Cys800 enhances the binding with the histone acetyltransferase p300, potentiating gene transcription [86,87] (Figure 1, upper panel) although the opposite has been also reported [88]. Furthermore, NO also protects HIF1α from proteasomal degradation because it impairs HIF1α binding with the Von Hippel–Lindau (VHL) protein and subsequent recruitment of the E3 ligase by inhibiting prolyl hydroxylases activity [89,90]. The impairment of ubiquitination and proteasomal-dependent degradation is a quite common mechanism by which nitrosylation regulates TFs activity. Nitrosylation of Cys77 of HDM2, the human homologue of mouse double minute-2, which resides at the interface of the interaction domain with p53, inhibits proteins binding and p53 proteasomal degradation [91]. This series of events results in p53 stabilization and enhanced activation [92] (Figure 1, upper panel). Direct p53 nitrosylation of Cys124 in skeletal muscle promotes its binding to the *ppargc1a* promoter, activating an antioxidant pathway and ensuring skeletal muscle cell homeostasis [93] (Figure 1, upper panel). Another well-known nitrosylated TF is NF-kB. In thyroid cells, nitrosylation of NF-kB subunit p65 on Cys38 leads to a repression of TSH-induced Na^+^/I^−^ symporter (NIS) gene expression due to NF-kB detachment from the strongly TSH responsive NIS upstream enhancer (NUE) between nucleotides 2264 and 2495 upstream the NIS proximal promoter [94] (Figure 1, upper panel). TSH is a NF-kB activator [95]. As TSH also induces NO production in thyroid cells by enhancing the transcription of NOS3, p65 nitrosylation might represent a feedback loop to control NF-kB activity in thyroid cells. Cys38 is also nitrosylated in respiratory epithelial cells and macrophages upon cytokines stimulation, a phenomenon that depends on NOS2 activity. Since NF-kB binds and activates the NOS2 promoter in the presence of cytokines, still NOS2-dependent nitrosylation of p65 represents a negative feedback loop to control the expression of genes downstream NF-kB signaling [96]. Some reports address a role to nitrosylation in ruling NF-kB p50 DNA binding activity. Indeed, it has been observed that nitrosylation of Cys62 inhibits the DNA binding capacity of p50 both in vitro and in vivo [97,98] (Figure 1, upper panel).

Another transcription factor undergoing nitrosylation is Myocyte Enhancer Factor 2 (MEF2), a member of a family of TFs which comprises MEF2A, B, and C. In α-synuclein mutant dopaminergic neurons, basal and/or toxin-induced nitrosative stress results in MEF2C nitrosylation of Cys39 and inhibition of the MEF2C/proliferator-activated receptor-g coactivator-1α (PGC1α) transcriptional axis, leading to mitochondrial dysfunction and apoptosis [99]. The same phenomenon accounts for the loss of DNA-binding ability of both MEF2A and MEF2C in cerebrocortical neurons. Nitrosylated MEF2C induces neuronal apoptosis by decreased binding to the *BCL-xL* promoter, whereas nitrosylated MEF2A impairs adult neurogenesis by loss of binding to pivotal neurogenesis-related genes [100] (Figure 1, upper panel).

Activator Protein -1 (AP-1), an ubiquitously expressed heterodimer constituted by c-jun and c-fos TFs, also undergoes nitrosylation. Both AP-1 subunits are regulated by nitrosylation, which inhibits their DNA binding [101,102,103] (Figure 1, upper panel). Specifically, c-fos is nitrosylated on Cys154 and c-jun on Cys272. Although most of the evidence on AP-1 nitrosylation has been obtained in vitro, some reports address a role of this AP-1-specific post-translational modification in vivo. Indeed, in neonatal mouse cardiomyocytes, nitrosylation of c-jun leads to a repression of tissue inhibitor metalloproteinase-3 (TIMP-3) expression, enhancing cardiomyocytes proliferation [104].

#### 2.2.2. S-Nitrosylation of Transcriptional Co-Regulators 

As well as TFs, chromatin remodeling enzymes, which act as transcriptional co-regulators, are exposed to nitrosylation when NO cellular concentration raise upon a variety of stimuli. Main outcomes of transcriptional co-regulators modifications are the impairment of their enzymatic activity and binding to other nuclear proteins. We shall focus only on the transcriptional impact of nitrosylation-dependent modifications of co-regulators although many of them exert also cytoplasmic roles [105,106]. The most well-known chromatin modifier modulated by nitrosylation is histone deacetylase 2 (HDAC2). The seminal work of Nott et al. was the first documented demonstration that HDAC2 is nitrosylated on Cys272 and Cys274. In neurons, nitrosylated HDAC2 does not lose its enzymatic activity; rather, it detaches from a series of promoters ruling the expression of genes playing pivotal role during neurogenesis, such as *Fos*, *Egr1*, *Vgf,* and *NOS1,* by inducing histone hyperacetylation at the corresponding chromatin loci (Figure 1, upper panel). At the functional level, HDAC2 nitrosylation in cortical neurons is important to promote dendritic growth, possibly allowing the recruitment of the cAMP response Element Binding Protein (CREB) at its cognate chromatin binding sites on gene promoters, ruling dendritic growth, and branching [107]. Furthermore, nitrosylation of HDAC2 positively affects the expression of 20 transcripts in the developing cortex. Among them, *Brahma* (*Brm*), a component of the Brm/Brg complex belonging to the SWitch/Sucrose Non Fermentable (SWI/SNF) family of ATP-dependent chromatin remodeling complex, appears in the developing brain at E15.5 together with HDAC2 and NOS2 and is essential for neuronal radial migration. This latter process strictly relies on the detachment of nitrosylated HDAC2 from the *Brm* promoter [108].

In skeletal muscle HDAC2, nitrosylation, occurring upon proper NO production, seems to be required for myotube formation and homeostasis. In fact, in dystrophic muscles, nitrosylation of HDAC2 has been indicated as partially responsible for the NO-dependent recovery of muscle morphology [109]. This phenomenon might depend on the de-repression of HDAC2 de-regulated genes, such as follistatin [110]. In this case, both global HDAC2 activity and displacement from chromatin are affected by nitrosylation. In a sub-population of embryonic stem cells, NOS3-dependent HDAC2 nitrosylation promotes its dissociation from Zeb1 transcriptional repressor, leading to the expression of mesendodermal genes and efficient production of cardiovascular precursors [111].

Nitrosylation of HDAC8 has been only demonstrated in vitro [112], and nitrosylation of other class I, II, and IV HDACs has not been documented so far. Sirtuins (class III HDACs) are also nitrosylated [113]. In physiological conditions, Sirt1, by deacetylating p53 and NF-kB [114,115], impairs their transcriptional activity and the activation of inflammatory and apoptotic processes. On the contrary, during inflammation, when NOS1 is activated and overproduces NO, nitrosylation inhibits Sirt1 activity by disrupting its ability to bind Zn^2+^, essential for the completion of sirtuins function (Figure 1, upper panel). Consequently, p53 and NF-kB are activated and induce the expression of inflammatory and apoptotic genes [116,117].

In induced pluripotent stem cells (iPSCs), nitrosylation of Metastasis Associated Family Member 3 (MTA3), which belongs to the Nucleosome Remodeling Deacetylase (NuRD) complex, accounts for DNA accessibility of pluripotency genes, such as Oct4, Nanog, and SOX2. This phenomenon relies on the loss of binding of SNO-MTA3 with HDAC2, which also participates to the formation of NuRD complex. Disruption of SNO-MTA3/HDAC2 interaction leads to an enrichment of acetylated and to a decrease of methylated histones in the promoter regions of *Oct4*, *Nanog,* and *SOX2* genes [118] (Figure 1, upper panel).

During transdifferentiation of BJ fibroblasts into endothelial cells, the Ring Finger Protein 1A (RING1A)—a component of the Polycomb repressor Complex 1 (PRC1)—is nitrosylated by NOS2 at Cys398 residue. Cys398 nitrosylation reduces RING1A binding to chromatin and decreases methylation of lysine (K) 27 on histone H3 de-repressing endothelial-specific gene promoters [119] (Figure 1, upper panel).

**Table 1:** Nuclear targets and impact of S-nitrosylation in physiology.

Nuclear Protein Target	Function	Physiological Outcome
**p53**	Increase of chromatin binding	Skeletal muscle homeostasis [93]
**MEF2**	Loss of chromatin binding	Impairment of adult neurogenesis Apoptosis of cerebrocortical neurons [99,100]
**AP-1**	Loss of chromatin binding	Cardiomyocytes proliferation [104]
**HIF-1α**	Enhanced interaction with acetyltransferases Protein stabilization	Angiogenesis [86,87,89,90]
**NF-kB**	Loss of chromatin binding	Modulation of thyroid hormone synthesis and inflammation [94,95,96,97,98]
**HDAC2**	Loss of chromatin binding Impairment of protein-protein interaction Inhibition of deacetylase activity	Neurogenesis Skeletal muscle homeostasis Mesendodermal differentiation [107,108,109,111,120]
**HDAC8**	Inhibition of deacetylase activity	Activation of transcription (demonstrated only in vitro) [112]
**Sirt1**	Inhibition of target proteins deacetylase activity	Inhibition of inflammation [116,117]
**MTA3**	Loss of protein-protein interactions	Pluripotency [118]
**RING1A**	Loss of chromatin binding	Transdifferentiation [119]

## 3. Role of S-Nitrosylation during Carcinogenesis 

The impact NO-and especially RNS-may have on DNA and chromatin architecture is particularly important in cancer cells, where NO metabolism might be deregulated. Indeed, some cell types lacking GSNOR and experiencing high levels of SNOs are prone to acquire a tumorigenic phenotype through an impairment of DNA damage-repair protein function by nitrosylation [121,122].

It is important to note that basal levels of nitrosylation are required for the maintenance of cellular homeostasis [123,124,125,126]. In cancer cells, the nitrosylation/de-nitrosylation process is unbalanced in favor of the generation of high quantities of SNO-proteins. This largely depends on the hypoxic environment promoted and sensed by the increasing tumor mass and inducing the generation of RNS [127]. An example of nitrosylation-guided carcinogenesis is the activation of membrane receptors and intracellular kinases upon the addition of SNO moieties, such as Epidermal Growth Factor Receptor (EGFR) [128], ras [129], src [130], and Akt [131]. All these events have a profound impact on transcriptional cancer cell reprogramming, leading to tumor progression and invasion. In colorectal cancer, nitrosylation of latent TGF-β binding protein 1 (LTBP1), favored by the formation of a macromolecular complex formed by phosphorylated 6-pyruvoyltetrahydropterin synthase (PTPS) and NOS1 in hypoxic conditions, leads to LTBP1 instability by increased ubiquitination and proteasome degradation. This, in turn, impairs TGF-β secretion and inhibition of cancer cell proliferation [132].

However, some nitrosylation events lead to apoptosis of cancer cells. In colorectal and breast tumor cells, nitrosylation of cIAP1, a member of the Inhibitor of Apoptosis (IAP) family of proteins [133], has been found to impair its TNF-α-dependent E3 ubiquitin ligase activity and degradation of receptor-interacting serine/threonine protein kinase 1 (RIP1). RIP1 degradation usually mediates TNF-α activation of NF-kB. Upon cIAP1 nitrosylation on Cys571 and Cys574, inhibition of RIP degradation switches a TNF-α survival program to an apoptotic one [134]. In lung cancer, nitrosylation of Cys51 and Cys172 of peroxiredoxin-2 (Prdx2)—an antioxidant enzyme that protects tumor cells from toxic level of H_2_O_2_—impairs the formation of Prdx2/Prdx2 homodimers, repressing its antioxidant activity. The increased levels of H_2_O_2_ boosts AMP-activated protein kinase (AMPK), which phosphorylates Sirt1 on Threo344, inhibiting its ability to bind and deacetylate either p53 or forkhead box protein O1 (FOXO1). p53-Enhanced acetylation leads to the occurrence of a p21-dependent apoptotic pathway. FOXO1 acetylation, together with NO-dependent Akt inhibition, induces nuclear retention of FOXO1 and activation of its pro-apoptotic targets *bim* and *puma* [135].

Genes encoding for mitogenic factors, TFs and chromatin regulators are well represented among NO-regulated transcripts in cancer cells [136,137]. Nitrosylation might modulate the activity of oncogenes, tumor suppressors, and chromatin remodelers indirectly by modifying upstream signal transduction molecules and directly, thus affecting carcinogenesis either negatively or positively. Indeed, we found a relatively high basal level of nitrosylated HDAC2 in glioblastoma stem cells cultured in the absence of NO donors, which are instead required for the generation of HDAC2-SNO in other cell systems (Salvatori L. and Illi B., unpublished). Table 2 summarizes nitrosylated protein targets discussed below.

### 3.1. S-Nitrosylation of TFs in Cancer

In general, nitrosylation of TFs results in reduced chromatin binding capacity and has a negative effect on tumor progression. A proteomic analysis in pancreatic ductal adenocarcinoma (PDAC) has identified 434 nitrosylated proteins. Among them, v-raf-1 murine leukemia viral oncogene homolog 1 (Raf-1), signal transducer and activator of transcription 1 and 3 (STAT1, 3), and retinoblastoma (RB) protein were found to be heavily nitrosylated [138]. Nitrosylation appears to be detrimental for STAT3-dependent oncogenic function in PDAC and also in multiple myeloma (MM) [139], where SNO-STAT3 shows decreased activity. In MM, nitrosylation inhibits the activity of STAT3 and NF-kB, leading to apoptotic cell death (Figure 1, lower panel). This occurs through a diminished expression of STAT3 and NF-kB anti-apoptotic target genes, such as *Pim2*, *Bcl-2*, *Bcl-_XL_*, and *Mcl-1* [139]. Nitrosylation inhibits NF-kB DNA binding in colon cancer cells [140] and decreases STAT3 phosphorylation and activation in head and neck squamous cell carcinoma (HNSCC) [141].

Moreover, nitrosylation of NF-kB, YY1, and Snail impairs EMT by blocking the transcriptional activity of this circuitry in prostate cancer cells (Figure 1, lower panel). Indeed, p50-SNO fails to translocate NF-kB to the nucleus and to activate YY1 and Snail. YY1-SNO has reduced transcriptional activity and fails to induce transcription of genes required for EMT, such as *vimentin* and *fibronectin* [142]. In parallel, NF-kB nitrosylation, promoted by NO high levels, might also impair tumor cell adhesion and extravasation into the vascular tree by inhibiting its binding to promoter regions of genes encoding adhesion molecules, such as *V-CAM1* and *ICAM1* [48,143].

S-nitrosylation-dependent inactivation of β–catenin/TCF4 transcriptional activity leads to growth inhibition of T-acute cell leukemia (T-ALL) Jurkat cells. In this case, nitrosylation of β–catenin results in its subsequent degradation and to the transcriptional repression of its target cyclin D1 [144]. In colon cancer cells, nitrosylation of β–catenin results in the dissociation of the β–catenin/TCF4 complex, inhibiting TCF-4 transcriptional activity [145] (Figure 1, lower panel).

One exception to the rule is represented by HIF1α. Indeed, it has been found that in murine tumors, Cys533 S-nitrosylation protects radiation-induced HIF1α, whose levels are increased by the release of stored HIF1α mRNAs from stress granules independently from hypoxia, from degradation in normoxic conditions [146]. Stabilized HIF1α-SNO still induces the expression of VEGF and other molecules to protect the tumor vessels from radiation-dependent cytotoxic damage. This has obvious clinical implications for the design of optimal strategies to counteract tumor resistance to radiation therapies. Consistently, inhibition of HIF1α nitrosylation by caveolin-1, a well-known NOS inhibitor [147], decreases HIF1α activity in a variety of cancer cells [148] (Figure 1, lower panel).

p53 is another TF whose nitrosylation may enhance protein stability. Indeed, nitrosative stress induced by metal nanomaterials provides p53 nitrosylation, increases p53 stability, and induces a pro-apoptotic pathway in lung cancer cells [149].

### 3.2. S-Nitrosylation of Transcriptional Co-Regulators in Cancer

Although very few reports document nitrosylation of chromatin remodeling enzymes in tumors, this could represent a highly frequent phenomenon ruling the cancer cell epigenome. Indeed, global changes in histone acetylation/methylation levels have been observed in several cancer cells [150] exposed to NO donors, which might reflect changes in the activity of chromatin remodelers. Globally, NO represses histone acetylation while enhancing methylation [151]. However, this effect might be histone-specific, as occurs in MDA-MB-231 triple-negative breast cancer cells, where the decrease in acetylation of K9 of H3 is accompanied by an increase, although minimal, of H3K27acetylation, whereas acetylation levels of other histone K residues (e.g., H3122ac) do not change. The distribution of acetylated/methylated H3 specifically changes upon NO exposure, with K9 acetylated H3 being enriched at those chromatin promoters corresponding to enhanced mRNA expression of related transcripts, such as the Ets-1 oncogene, Fos, Jun, and VEGFA. In parallel, H3K9 methylation is lost from chromatin domains ruling the expression of genes important for tumor progression and spread (e.g., MMP-1 and 10) [150].

Another indirect evidence that chromatin remodelers might be regulated by nitrosylation in cancer is the design of compounds constituted by histone deacetylase inhibitors conjugated with NO donors [152,153], which may exert potent apoptotic and anti-tumor activities [152].

A direct effect of nitrosylation on chromatin remodeling enzymes in tumors has been observed for class I HDACs, specifically for HDAC2. In melanoma cells, NOS1-dependent impairment of interferon-α response relies on Cys272 and Cys274 HDAC2 nitrosylation. This modification promotes HDAC2 dissociation from STAT1, enhancing acetylation of K16 on H4 and deregulating interferon-α-stimulated genes (ISGs). Indeed, contrary to other acetylated histones, H4K16ac is a mark of gene silencing, and its deacetylation is important for RNA polymerase II (RNAP II) recruitment at gene promoters. Lack of H4K16 deacetylation by HDAC2 nitrosylation leads to the loss of ISGs expression in melanoma cells. This phenomenon is important for lung metastasization of melanoma, as mice injected with melanoma cells carrying non-nitrosylable form of HDAC2 (C272A/C274A) do not develop lung metastases [154] (Figure 1, lower panel).

**Table 2:** Nuclear targets and impact of S-nitrosylation in tumor cells.

Nuclear Protein Target	Function	Tumor
**STAT3**	Decrease of STAT3 activity, increase of apoptosis Decrease of STAT3 phosphorylation and activation	Multiple myeloma Head and neck carcinoma [139,141]
**p53**	Increase of stability	Lung cancer [149]
**NF-kB**	Decrease of NF-kB activity, increase of apoptosis Inhibition of NF-kB DNA binding Impairment of NF-kB nuclear translocation, prevention of EMT Impairment of tumor cell adhesion and extravasation	Multiple myeloma Colon cancer Prostate cancer Breast cancer and Melanoma [139,140,142,143]
**YY1**	Prevention of YY1 activation, impairment of EMT	Prostate cancer [142]
**Snail**	Prevention of Snail activation, impairment of EMT	Prostate cancer [142]
**β-catenin**	Inactivation of β–catenin/TCF4 transcriptional activity, repression of cyclin D1, growth inhibition	Leukemia Colon cancer [144,145]
**HIF-1 α**	Protein stabilization, induction of VEGF expression	Breast cancer [146,147,148]
**HDAC2**	Enhancement of H4 acetylation, inhibition of interferon-α-stimulated genes expression	Melanoma [154]

## 4. Conclusions: Gaps and Future Directions

S-nitrosylation is emerging as a major NO-dependent post-translational mechanism ruling gene expression. Its reversible nature makes nitrosylation a versatile tool by which NO might control gene expression programs in health and disease. Nevertheless, despite the great amount of information acquired regarding nitrosylation-dependent regulation of signalling molecules [128,129,130,131,155,156] and transcription factors (see Section 3.1.) pivotal to cancer progression, there is lack of knowledge about the control of transcriptional co-regulators activity by the addition of SNO moieties in tumors. Indeed, to our knowledge, besides HDAC2, the only enzyme controlling acetylation/deacetylation of proteins regulated by nitrosylation in tumor cells is the class IIb HDAC6 [105]. HDAC6 is, however, a cytoplasmic molecule affecting the acetylation levels of α-tubulin and assembly/disassembly of microtubules together with Hsp90 activity and aggresomal formation [157,158,159]. This is surprising since several lines of evidence suggest a role of nitrosylation in modulating the activity of chromatin remodelers within the nucleus of cancer cells. Indeed, global changes in the histone modification profile characterize breast and lung cancers exposed to NO [136,150]. Nevertheless, the volume of scientific information related to nitrosylation-dependent epigenetic control of biological processes in non-tumor cells strongly exceeds what is actually known for cancer (Figure 1).

Furthermore, it has not been determined yet whether nitrosylation might affect other important chromatin modulators, such as histone methyltransferases and/or de-methylases, DNA methyltransferases (DNMTs), and the SWI/SNF family of ATP-dependent remodelers, regardless the cell type. To the best of our knowledge, the only histone methyltransferase indirectly regulated by nitrosylation is suppressor of variegation 3–9 homolog 1 (SUV39H1). In fact, in neurons, NO-dependent nitrosylation of GAPDH/seven in absentia (Siah) homolog complex promotes SUV39H1proteasomal degradation and neurotrophin- and CREB-dependent neurites outgrowth [160]. A role for nitrosylation in regulating the activity of histone de-methylase, such as LSD1 and the JumonjiC (JMJC) family of de-methylases, is far from being determined, whilst the action of NO in inhibiting the activity of JMJC and in regulating the expression of other de-methylases has been demonstrated [161].

Several observations point to a role of NO in regulating DNMTs and inducing DNA CpG islands methylation, but how NO accomplishes this activity must still be elucidated [162,163]. The regulation of the SWI/SNF complex by nitrosylation is, to our knowledge, still a dark matter, whereas molecular circuitries involving SWI/SNF molecules responsible for NO production in a variety of cell systems have been discovered [164,165].

The balance between nitrosylation/de-nitrosylation is a delicate process required to ensure a proper cellular homeostasis. Deregulated de-nitrosylation of SNO-proteins might be as important as RNS-dependent hyper-nitrosylation in the onset of different human diseases, including cancer. Nevertheless, many aspects have still to be elucidated, and the possible targeting of nitrosylation/de-nitrosylation processes to control the pathogenesis and the progression of human diseases is still in its infancy. Indeed, we are aware that although S-nitrosylation is clearly responsible of many NO-regulated biological outcomes both in physiological and pathological contexts, it is not the unique mechanism of action of NO.

The decrease of GSNOR in solid tumors [166,167] is consistent with the increase of SNO-protein species whose activity might be either boosted or inhibited by nitrosylation. According to the tumor type, protein nitrosylation might specifically affect tumor biology, and proteomics of tumor SNO-proteins may provide a patient-specific footprint suitable for diagnostic and prognostic purposes. Therefore, in the era of personalized medicine, the fine tuning of nitrosylation might represent an unexplored field that could provide a novel therapeutic opportunity for the treatment of specific cancers.

## Data Availability

Not applicable.
